# 

**Published:** 1920-10-30

**Authors:** 


### Matrons' and Sisters' Appointments.

Infectious Hospital, Bishop Auckland.—Miss Hilda Todd has been appointed matron. She was trained at Crumpsall Infirmary, Manchester, and has recently been ward sister at the Poor-Law Infirmary, Bishop Auckland.

Warrington Infirmary and Dispensary.—Miss A. Knox has been appointed sister. She was trained at the Chesterfield Royal Hospital and at Dewsbury Fever Hospital, and has been staff nurse at Bradford Royal Infirmary.

Florence Nightingale Hospital, Bury.—Miss E. J. Whitehead has been appointed sister. She was trained at the Rochdale Hospital and at Farnworth Fever Hospital, and has been superintendent at the Red Cross Hospital, Wigan.

Sunderland Mental Hospital, Ryhope.—Miss I. M. Fairgrieve has been appointed matron. She was trained at the Royal Infirmary, Manchester, and the City Fever Hospital, Edinburgh, and has been assistant matron at the Crichton Royal Institution, Dumfries.

City Mental Hospital, Gosforth, Newcastle-on-Tyne.—Miss Beatrice Butler Allbutt has been appointed assistant matron. She was trained at Queen's Hospital, Birmingham, and has been assistant matron at the District Mental Hospital, Dundee, and at Craig House, Morningside, Edinburgh.

Ellesmere Port and District Cottage Hospital, near Birkenhead.—Miss L. E. Barrett has been appointed sister. She was trained at the General Hospital, Darlington, and at the Isolation Hospital, Warrington. Later she became staff nurse at the Brook Fever Hospital, Woolwich, and then served on the private staff of Westminster Hospital. She was staff nurse and sister under the Q.A.I.M.N.S.R. in France, and with the Army of Occupation in Germany. Miss Barrett is a member of the College of Nursing.

### Gifts and Bequests.

The Royal Midland Counties Hospital for Incurables, Leamington, has been left £100 by Mr. Edward Parry.

Nottingham General Hospital and Nottingham Eye Hospital will each receive £50 under the will of Mr. Samuel Henry Sands.

Sir William and Lady Lister have sent a donation of £5,000 to the Royal London Ophthalmic Hospital for the purpose of starting an ophthalmic convalescent home.

Major-General W. E. Marsland, of Hove, has left £100 to the Sussex County Hospital, and £200 each to the Hove Dispensary, the Brighton Hospital for Women, and the Alexandra Hospital for Children.

Subject to certain life interests, £1,000 of the estate of Dr. George James Robertson, of Old Meldrum, Aberdeen, has been willed to the Aberdeen University in trust for the advancement and practice of surgery.

